# Obesity Is a Thrombotic Risk Factor in Pregnant Women

**DOI:** 10.3390/jcm14155310

**Published:** 2025-07-28

**Authors:** Daria Korolova, Andrea Suranyi, Anastasiia Pavlenko, Abel T. Altorjay, Svitlana Zhuk, Iryna Us, Yurii Melnyk, Volodymyr Chernyshenko, Sandor G. Vari

**Affiliations:** 1 Palladin Institute of Biochemistry of NAS of Ukraine, 01054 Kyiv, Ukraine; 2 Albert Szent–Györgyi Medical School, University of Szeged, H-6720 Szeged, Hungary; 3 The Shupyk National Medical Academy of Postgraduate Education, 04112 Kyiv, Ukraine; 4 International Research and Innovation in Medicine Program, Cedars-Sinai Medical Center, Los Angeles, CA 90048, USA

**Keywords:** pregnancy, obesity, platelets, thrombosis, soluble fibrin, D-dimer, fibrinogen

## Abstract

**Background/Objectives**: Pregnancy is associated with increased procoagulant conditions, and when combined with obesity, it can elevate the risk of thrombosis. The study aims to assess thrombosis risk markers during pregnancy in relation to obesity. **Methods**: Somatically healthy women aged 18–42 years with spontaneous pregnancies who did not receive specific antithrombotic treatment were enrolled in the study (n = 97). The participants were divided into groups based on pregestational BMI: the first group consisted of patients who had a BMI ≤ 25 (n = 42), and the second group consisted of patients who were overweight (BMI > 25) and obese (BMI > 30) (n = 55). The control group comprised healthy, non-pregnant, non-obese women (n = 10). **Results**: Fibrinogen levels, elevated during pregnancy, were higher in the II and III trimesters, with gestational period having a greater influence than BMI. Moderate D-dimer accumulation was observed regardless of obesity, but higher levels were seen in obese women during the III trimester, indicating the dissolution of intravascular fibrin deposits. Soluble fibrin was significantly higher in obese and overweight women during the II trimester and elevated in both groups during the III trimester, correlating with D-dimer accumulation and indicating thrombus formation. A decrease in platelet aggregation ability was observed correlating with D-dimer and soluble fibrin patterns. **Conclusions**: A significant accumulation of thrombosis risk markers was observed in the III trimester compared to the II, occurring earlier in obese and overweight pregnant women and indicating a higher risk of thrombotic complications in obesity.

## 1. Introduction

Obesity during pregnancy is a significant global health issue. A systematic review of over 49 million pregnancies (2010–2019) found that 16.3% of pregnant women were classified as obese, with nearly 44% being overweight or obese combined [1]. This is associated with increased risks for both the mother and the baby. For the mother, obesity can lead to complications like gestational diabetes, preeclampsia, and an increased likelihood of needing a cesarean section [2,3]. For the baby, risks include being born with a higher birth weight, which can lead to delivery complications, as well as an increased risk of childhood obesity and other health issues later in life [4]. Obesity is a risk factor for venous thromboembolism (VTE) in pregnancy, and the risk is higher with increasing obesity [5,6]. Being overweight (BMI 25.0–29.9), too, is a weak risk factor for pregnancy-related VTE and is extremely common, with a prevalence within the childbearing population of almost 50% [7].

Although the precise mechanism behind the increased risk of VTE in obese women is unclear, previous studies have hypothesized that it could be associated with physical factors, such as blood flow and adiposity. Reduced or stagnant blood flow promotes the accumulation of clotting factors and platelets, creating a prothrombotic environment that increases the risk of venous thrombus formation. As for adipose tissue, it could be associated with the pro-thrombotic state found in obesity, which influences hemostasis, coagulation, and fibrinolysis [8]. The various mechanisms by which obesity may cause arterial and venous thrombosis include: the actions of adipocytokines from adipose tissue (e.g., leptin and adiponectin); increased activity of the coagulation factors (fibrinogen, factor VII, factor VIII, von Willebrand factor) and decreased activity of the fibrinolytic system (plasminogen activator inhibitor [PAI]); increased inflammation (tumor necrosis factor [TNFα], interleukin-6 [IL-6]); increased oxidative stress and endothelial dysfunction; disturbances of lipids and glucose tolerance in association with the metabolic syndrome [9].

Understanding the characteristics of changes in hemostasis in women with obesity during pregnancy will contribute to a more effective prediction of the thrombotic risk in such women so as to be able to make a decision on timely antithrombotic prophylaxis [10]. Routine laboratory methods are insensitive to hypercoagulation in obese pregnant women [11].

On the other hand, today, as a result of the lack of clinical protocols and the unanimity of the results of large-scale studies, there is no clear understanding of the concept of thrombophilia [12]. Although the term thrombophilia is quite well known in obstetric and gynecological practice, its meaning often has quite different interpretations and, accordingly, different vectors of preventive and therapeutic measures. In fact, the question of what thrombophilia is remains relevant—it is a disease that must be treated, or a state of thrombotic readiness that precedes a thrombotic event or a risk factor that is realized under a certain set of circumstances. At the same time, it should be noted that in the world community, there is no unambiguous definition of the concept of thrombophilia today, and any association or scientific group of authors studying this issue brings its own certain innovations to the understanding of this term. Also, today there is no common opinion, a single consensus, or a universal agreement as regards which tests should be included in thrombophilic screening [13].

*The purpose of the study*: increasing the effectiveness of diagnostics of thrombophilic blood changes to assess thrombotic risks in pregnant women with obesity.

## 2. Materials and Methods

### 2.1. Cohorts

Women aged 18–42 years with no somatic health issues with spontaneous pregnancies that did not receive specific antithrombotic treatment were enrolled in the study (n = 106) at the Department of Obstetrics & Gynecology, University of Szeged.

Inclusion criteria: patients aged 18–42 years with singleton pregnancies conceived spontaneously, without severe pregnancy complications or significant extragenital pathology. Exclusion criteria: age less than 18 or more than 45 years, multiple pregnancy, pregnancy resulting from IVF, taking antiplatelet drugs during pregnancy, severe extragenital pathology, chromosomal pathology, fetal malformations, gestational hypertension, gestational diabetes mellitus. Each trimester group consisted of different individuals; no patient was assessed more than once across trimesters.

Following data about patients were collected: date of examination, LMP last menstrual period, gestational age at sampling, accompanied diseases, OGTT 0’, OGTT 120’, weight (kg), height (cm), weight gain during pregnancy (kg), preBMI (pregestational body mass index), outcome (sc/pvn), complications (indication for termination), gestational age at delivery (w), date of delivery, BW (g), gender, APGAR 1’, APGAR 5’, APGAR 10’.

Gestational age at sampling and BMI before gestation were selected as the main parameters to analyze. So, studied patients were divided into groups according to BMI: first group had BMI ≤ 25 (n = 42); second group had BMI > 25 (n = 55); obesity is indicated by a BMI of more than 30, overweight by a BMI ranging between 25 and 29.9. Data received during the II trimester and during the III trimester of gestation were compared.

Venous blood of healthy, non-pregnant volunteers (n = 10) with normal BMI who had not taken any medication for 7 days was used as a control probe.

All women provided oral and written informed consent for their inclusion in the study.

### 2.2. Materials

Monoclonal antibodies I-3C, II-4, and III-3B, thrombin-like enzyme from snake venom, were obtained in Palladin Institute of Biochemistry of NAS of Ukraine (Kyiv, Ukraine) [14]; ADP was purchased from Sigma–Aldrich (St. Louis, MO, USA).

### 2.3. Blood Plasma Sampling

Venous blood was taken for testing using vacuum systems in tubes with 3.8% sodium citrate using 19G sterile needle. Platelet-poor plasma was obtained by centrifugation of blood at 400× *g* for 20 min at 25 °C.

### 2.4. Preparation of Platelet-Rich Blood Plasma (PRP)

PRP was obtained by centrifugation of blood at 160× *g* for 30 min at 25 °C. PRP with the platelet count from 150 to 350 cells/μL can be found above the red phase of blood cells [15].

### 2.5. Fibrinogen

Fibrinogen concentration in the blood plasma was determined by the modified spectrophotometric method. Blood plasma (0.2 mL) and PBS (1.7 mL) were mixed in a glass tube. Coagulation was initiated by the addition of 0.1 mL of thrombin-like enzyme from the *Aghistrodon halys halys* snake venom (1 NIH/mL) to prevent fibrin cross-linking. Mixture was incubated during 30 min at 37 °C. The fibrin clot was removed and resolved in 5 mL of 1.5% acetic acid. The concentration of protein was measured using spectrophotometer POP (Optizen, Daejeon, Korea) at 280 nm (ε = 1.5) [16].

### 2.6. Soluble Fibrin

Soluble fibrin was detected using sandwich ELISA with monoclonal antibodies produced in Palladin Institute of Biochemistry of NAS of Ukraine. Fibrin-specific monoclonal antibody I-3C was used as catch-antibody. Biotinilated monoclonal antibody II-4d that has an epitope at the NH_2_-terminal fragment of γ-chain of D-region of fibrinogen molecule was used as a tag-antibody. Optical density was measured at 450 nm using multiplate reader RT 2100C (Rayto, Shenzhen, China) [17].

### 2.7. D-dimer

D-dimer was detected using sandwich ELISA as described above for soluble fibrin with modification. Biotinilated D-dimer-specific monoclonal antibody III-3B that has an epitope at the NH_2_-terminal fragment of Bβ-chain of D-region of fibrin(ogen) produced in Palladin Institute of Biochemistry of NAS of Ukraine was used as the catch-antibody [18].

### 2.8. Platelet Aggregation

Platelet aggregation was measured based on changes in the turbidity of human PRP. In a typical experiment, 250 μL of PRP was incubated with 25 μL of 0.025 M CaCl_2_ and 25 μL of 12.5 μM ADP at 37 °C. Aggregation was monitored for 10 min using the aggregometer Solar 2110 (Solar–Techical service, Kharkiv, Ukraine) [19].

### 2.9. Statistics

Statistical data analysis was performed using the Kruskal–Wallis test. This test is a statistical test used to compare two or more groups for a continuous or discrete variable. Post-hoc analysis was performed according to the Mann–Whitney U test. This nonparametric test allows two groups to be compared without making the assumption that values are normally distributed. Social Science Statistics service was used for analysis (https://www.socscistatistics.com/, accessed September 2024–May 2025). All assays were replicated three times. Results are presented as boxplot diagrams with median, maximum, minimum values, and interquartile range. Data were considered significant when *p* < 0.05.

## 3. Results

### 3.1. Characterization of Selected Cohorts

The general characteristics of the patient are presented in Table 1. There is no significant difference between pregnant women who had a BMI < 25 kg/m^2^ (considered as normal) and those who had a BMI > 25 kg/m^2^ (overweight and obese). This can be explained by the strict exclusion criteria that were applied. Basically, we selected generally healthy pregnant women of different weights: normal, overweight, and obese.

This allowed us to compare the main parameters of the blood coagulation system between the two groups that differed only on the basis of body weight. As such, the following parameters were selected: fibrinogen, D-dimer, soluble fibrin, and platelet reactivity.

### 3.2. Fibrinogen

Under the influence of thrombin, fibrinogen is converted into fibrin, leading to the formation of fibrin fibers that constitute the core of a blood clot preventing blood loss. Beyond its role in thrombus formation, fibrinogen plays crucial roles in reparative and inflammatory processes. Its concentration increases exponentially during inflammation, correlating with the risk of intravascular thrombus formation [15].

**Figure 1:**
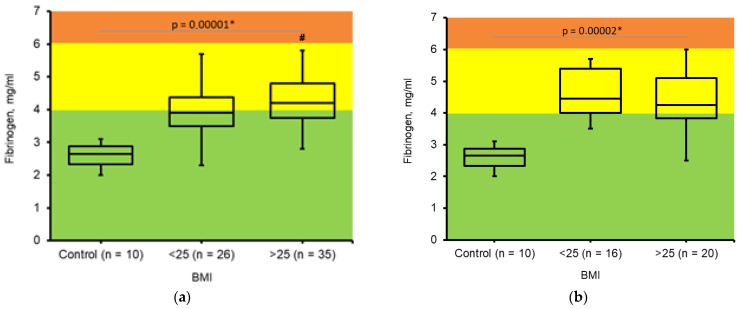
Changes in *fibrinogen* concentration in pregnant women. (**a**). Determined during II trimester of pregnancy; BMI ≤ 25 (n = 26); BMI > 25 (n = 35). (**b**). Determined during III trimester of pregnancy; BMI ≤ 25 (n = 16); BMI > 25 (n = 20). Control—healthy, non-pregnant, non-obese women (n = 10). The Kruskal–Wallis test was used to analyze results. * Results were assumed to be significant when *p* < 0.05. #—significant difference between two experimental groups according to Mann–Whitney U test.

In healthy individuals, the concentration of fibrinogen in blood plasma typically ranges from 2.0 to 3.5 mg/mL [20]. However, in various pathological conditions, fibrinogen levels can fluctuate between 1 mg/mL and 10 mg/mL (Figure 1).

To accurately measure fibrinogen concentration in the bloodstream, it is advisable to employ a spectrophotometric method using a thrombin-like enzyme (such as Reptilase, Ancistron, or Ancrod). This method involves converting all fibrinogen in the blood plasma into a fibrin clot, which is then dissolved in acetic acid for concentration determination using a spectrophotometer.

Concentration of fibrinogen was increased in some of the pregnant women in the II trimester of gestation (Figure 1a) independently of BMI. However, in the III trimester of gestation almost all studied women had elevated fibrinogen concentrations (Figure 1b). We considered fibrinogen as a non-specific parameter that is changed during pregnancy independently of body weight.

### 3.3. D-dimer

The D-dimer test is a mandatory component of diagnostic algorithms for conditions associated with intravascular thrombus formation. However, as a product of hydrolysis of stabilized fibrin, D-dimer cannot detect the potential for thrombus formation; it only reflects the consequences of thrombus formation. Therefore, D-dimer should be considered a post-thrombotic marker.

In healthy individuals, D-dimer concentrations in blood plasma do not exceed 100 ng/mL, although this value can vary depending on the specific test employed—up to 200 ng/mL for some variants [21].

The determination of D-dimer is based on solid-phase immunoenzymatic analysis using a monoclonal “catch” antibody specific to D-dimer (III-3B) and a monoclonal “tag” antibody specific to fibrinogen and its derivatives (II-4d).

**Figure 2:**
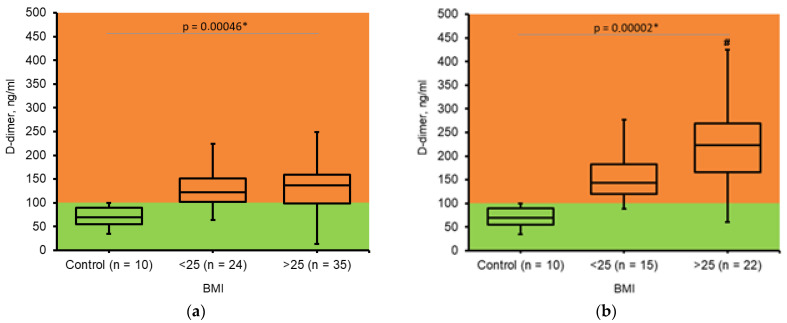
Changes in *D-dimer* concentration in pregnant women. (**a**). Determined during II trimester of pregnancy; BMI ≤ 25 (n = 24); BMI > 25 (n = 35). (**b**). Determined during III trimester of pregnancy; BMI ≤ 25 (n = 15); BMI > 25 (n = 22). Control—healthy, non-pregnant, non-obese women (n = 10). The Kruskal–Wallis test was used to analyze results. * Results were assumed to be significant when *p* < 0.05. #—significant difference between two experimental groups according to Mann–Whitney U test.

During the II trimester we noticed the slight elevation of D-dimer concentration in both groups (Figure 2a). However, overweight and obese pregnant women demonstrated excessive accumulation of D-dimer during the III trimester of gestation (Figure 2b). It indicated the formation and fibrinolysis of fibrin deposits in obese pregnant women preferentially; this must be an object of caution and constant monitoring.

### 3.4. Soluble Fibrin

**Figure 3:**
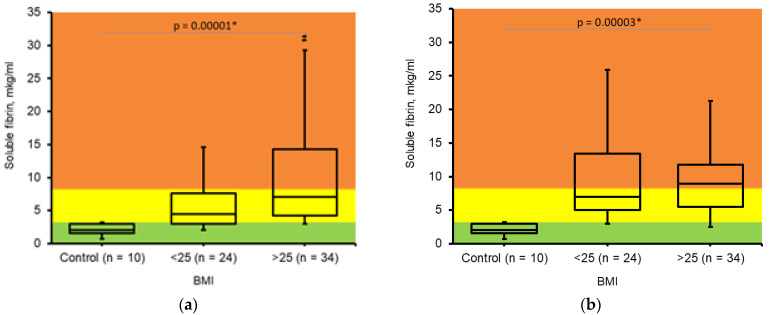
Changes in *soluble fibrin* concentration in pregnant women. (**a**). Determined during II trimester of pregnancy; BMI ≤ 25 (n = 24); BMI > 25 (n = 34). (**b**). Determined during III trimester of pregnancy; BMI ≤ 25 (n = 15); BMI > 25 (n = 22). Control—healthy, non-pregnant, non-obese women (n = 10). The Kruskal–Wallis test was used to analyze results. * Results were assumed to be significant when *p* < 0.05. #—significant difference between two experimental groups according to Mann–Whitney U test.

Soluble fibrin is a complex of fibrin oligomers and fibrinogen that circulates freely in the bloodstream. The accumulation of soluble fibrin indicates the presence of active thrombin, reflecting a dynamic imbalance in the hemostasis system. An increase in soluble fibrin concentration serves as an early prognostic indicator of intravascular coagulation activation, making its measurement crucial for preventing thrombus formation (Figure 3).

In healthy individuals, soluble fibrin concentration in blood plasma does not exceed 3 μg/mL. A concentration above 6 μg/mL suggests a significant risk of intravascular thrombus formation. The determination of soluble fibrin utilizes a solid-phase immunoenzymatic analysis with a monoclonal “catch” antibody specific to fibrin desAB (I-3C) and a monoclonal “tag” antibody specific to fibrinogen and its derivatives (II-4d).

Overweight and obese pregnant women demonstrated higher concentration of soluble fibrin during the II trimester of gestation (Figure 3a). Soluble fibrin accumulation was detected in both groups during the III trimester of gestation, indicating an overall increase in prothrombotic conditions (Figure 3b).

### 3.5. Platelet Aggregation

Platelets are the main cellular component of hemostasis that, being activated, interact with a polymerized fibrin three-dimensional network, forming the ‘body’ of a blood clot. Also, activation and degranulation of platelets lead to the release of growth factors and cytokines. So, the functional conditions of platelets are an important factor in intravascular thrombus formation.

In this study, we assessed platelet function by measuring platelet aggregation induced by ADP. We estimated the rate (maximal turbidity) and the speed of platelet clot formation.

Methods assessing platelet-derived factors, including counting the number of platelets and estimating their volume, have the limitations of not assessing the activity of cells. On the other hand, aggregometry allows us to observe directly how effective the platelet clot formation is in the platelet-rich plasma of selected patients.

**Figure 4:**
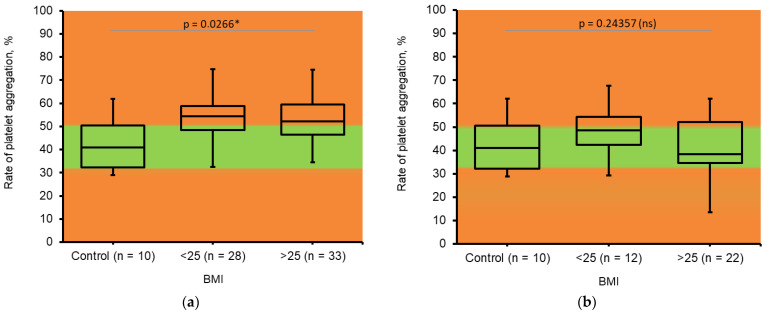
Changes in *rate* of platelet aggregation in pregnant women. (**a**). Determined during II trimester of pregnancy; BMI ≤ 25 (n = 28); BMI > 25 (n = 33). (**b**). Determined during III trimester of pregnancy; BMI ≤ 25 (n = 12); BMI > 25 (n = 22). Control—healthy, non-pregnant, non-obese women (n = 10). The Kruskal–Wallis test was used to analyze results. * Results were assumed to be significant when *p* < 0.05.

**Figure 5:**
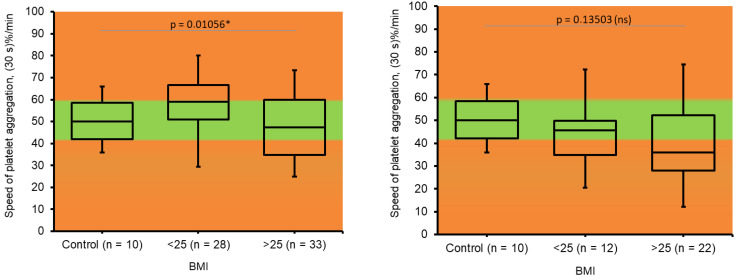
Changes in *speed* of platelet aggregation in pregnant women. (**a**). Determined during II trimester of pregnancy; BMI ≤ 25 (n = 28); BMI > 25 (n = 33). (**b**). Determined during III trimester of pregnancy; BMI ≤ 25 (n = 12); BMI > 25 (n = 22). Control—healthy, non-pregnant, non-obese women (n = 10). The Kruskal–Wallis test was used to analyze results. * Results were assumed to be significant when *p* < 0.05.

The overall tendency of an increase in platelet reactivity that was detected in pregnant women during II trimester of gestation is typical for pregnancy (Figure 4a and Figure 5a). Interestingly, a slight decrease in the rate and speed of platelet aggregation in the III trimester compared to the II trimester of gestation was determined in this study (Figure 4b and Figure 5b).

## 4. Discussion

Fibrinogen level elevation, typical for pregnant women, was observed in the II and III trimesters. Fibrinogen level did not depend on BMI. Fibrinogen levels undergo statistically significant changes during pregnancy and increase with increasing gestational age [22].

Moderate accumulation of D-dimer was observed in the II trimester of pregnancy in both groups of patients. However, in the III trimester, obese women demonstrated significantly higher concentrations of D-dimer, indicating fibrin formation and the activation of intravascular fibrinolysis [11].

The obtained results may indicate that the source of D-dimer may be extravascular deposits of fibrin [23]. And if we consider obesity as one of the forms of chronic inflammation, then obese women have higher levels of such fibrin.

Soluble fibrin concentration was significantly higher for overweight and obese women in the II trimester. Both obese and non-obese women had elevated concentrations of soluble fibrin in the III trimester. This was correlated with the accumulation of D-dimer and indicated the intravascular clotting system activation.

Soluble fibrin indicated the risk of thrombosis in obese women starting from the II trimester as an early marker of the danger of intravascular blood clotting. The soluble fibrin-based prediction during the earlier gestational period was proved by D-dimer accumulation in obese women in the late gestational period.

The majority of existing studies during pregnancy have demonstrated an increase in the aggregation activity of platelets, both spontaneous and induced, with most aggregation reagents [24,25]. We also detected the increase in platelet reactivity in pregnant women during the II trimester of gestation.

Also, we found a decrease in platelet reactivity in both obese and non-obese women during the III trimester of gestation compared to the II trimester. It can be assumed as the compensatory mechanism to balance the procoagulant shift is protein hemostasis. The above-mentioned changes in platelet reactivity were found earlier in obese women and correlate to the situation with D-dimer and soluble fibrin.

**Strengths and Limitations of the Study.** This study provides insight into potential pro-thrombotic mechanisms during pregnancy, particularly those associated with obesity. Although we did not specifically focus on clinical thrombotic events, as is common in much of the existing literature, we analyzed cellular and molecular markers to elucidate the intravascular processes that may contribute to clot formation. For this purpose, we used several specific tests, which are described and referenced in the Methods section. However, these may differ from the diagnostic assays commonly used in standard hemostasis laboratories. Therefore, the potential inability of other researchers to reproduce some of the data could be considered a limitation of the study. The main limitations of the study are the relatively small sample size and the absence of follow-up data on obstetric outcomes.

## 5. Conclusions

Soluble fibrin and D-dimer paired determination was shown to be a prospective diagnostic approach for the prognosis of the risk of thrombosis in pregnant women during gestation. The obtained data indicate the need to consider obesity as one of the additional risk factors for VTE during pregnancy. Also, our experience once again indicates the need to take into account body weight when prescribing antithrombotic prophylaxis during pregnancy.

## Figures and Tables

**Table 1:** General characteristics of the examined pregnant women. OGTT—Oral glucose tolerance tests, BW—birth weight, BMI—body mass index.

	BMI < 25 kg/m^2^		BMI > 25 kg/m^2^	
Trimester	II (n = 28)	III (n = 16)	II (n = 37)	III (n = 25)
Gestational age at sampling	21.04 ± 3.87	32.94 ± 2.11	21.16 ± 3.68	33 ± 3.71
Gestational age at delivery (w)	38.1 ± 3.79	38 ± 3	38.24 ± 1.05	38.84 ± 1.62
Weight gain during pregnancy (kg)	11.79 ± 4.48	12.69 ± 4.16	11.38 ± 7.3	12.44 ± 6.42
BW (g)	3076.43 ± 876.92	3401.88 ± 622.9	3317.06 ± 489.78	3365.56 ± 559.35
OGTT 0	4.24 ± 0.24	4.16 ± 0.27	4.34 ± 0.43	4.39 ± 0.4
OGTT 120	5.13 ± 1.3	4.63 ± 0.86	4.89 ± 1.3	4.88 ± 1.29

## Data Availability

The data can be accessed by email from the authors.

## Abbreviations

The following abbreviations are used in this manuscript:

VTE	Venous thromboembolism
BMI	Body mass index
PRP	Platelet rich blood plasma
